# What Promotes the Happiness of Vacationers? A Focus on Vacation Experiences for Japanese People During Winter Vacation

**DOI:** 10.3389/fspor.2022.872084

**Published:** 2022-05-26

**Authors:** Atsushi Kawakubo, Takashi Oguchi

**Affiliations:** ^1^Department of Psychology, Faculty of Humanities, Saitama Gakuen University, Kawaguchi, Japan; ^2^College of Contemporary Psychology, Rikkyo University, Tokyo, Japan

**Keywords:** wellbeing, vacation, benefits of vacation, travel, recovery experiences, Japanese sample

## Abstract

Several studies on tourism have examined the effects of vacation and travel on individuals' wellbeing. However, relatively little is known about the underlying psychological factors and mechanisms. Therefore, this study aimed to investigate the effects of a winter vacation on individuals' wellbeing. A total of 507 participants (255 men and 252 women) completed three questionnaires at three different time points. The questionnaires comprised psychological scales and items to seek demographic information so that the changes in their wellbeing could be assessed. The results revealed that people who traveled had higher subjective wellbeing than those who did not. Moreover, out of the four elements of the recovery experience, mastery was the only one influenced subsequent subjective wellbeing. The findings suggest that it is crucial to take vacations and to savor recovery experiences while off work. In particular, experiencing new and challenging events during a vacation was the most significant predictor of vacationers' subsequent wellbeing. Our results clarify what type of vacation is most effective for wellbeing. The results can help tourism practitioners manage their customers' experiences better during their vacations, and these efforts will arguably contribute not only to the wellbeing of vacationers but also to future company growth.

## Introduction

Wellbeing is one of the buzzwords of the decade and is omnipresent in almost all discourses related to daily human routines (Smith and Diekmann, 2017). The study of quality of life (QOL) and wellbeing grew from the Social Indicators Movement, which emphasizes the development of theories, research, and standards to track nations' social health (Sirgy, 2019). Social health includes having low-stress levels, and books are continuously being written on its methods to promote happiness through stress mitigation.

### Positive Influences of Vacation and Travel on Wellbeing

What can be done to reduce that stress when someone feels very stressed? One way to reduce the everyday stress that impairs wellbeing is to take a vacation. Various studies have identified the positive effects of taking and experiencing a vacation. Westman and Eden (1997) found that vacations could supplement social resources, thus decreasing work stress and making time to acquire new social resources. Further, vacations may provide unique opportunities with restorative effects, such as physical activity and social contact with family and friends (Gump and Matthews, 2000). As Dahlgren et al. (2005) demonstrated, a temporary absence from work may be needed for complete recovery from work-related fatigue. Furthermore, vacation improves wellbeing and leads to personal growth and fulfillment: “people can enjoy quality time among families and friends and times/activities that are undertaken are often linked to personal growth and fulfillment” (McCabe and Johnson, 2013, p. 61).

One of the main activities during vacations is travel. Newman et al. (2014) proposed five core psychological mechanisms that leisure potentially triggers to promote subjective wellbeing: detachment-recovery, autonomy, mastery, meaning, and affiliation. These psychological mechanisms promote leisure-specific subjective wellbeing, which enhances global wellbeing. Additionally, there was only one specifically mentioned leisure activity described in all five of the psychological mechanisms: travel (Loveday et al., 2018). Travel during vacations has been generally regarded as beneficial for individuals' physical and mental health (Chen et al., 2016). Researchers measured individuals' perceived health and wellness before and after leisure travel and then assessed the effects of tourism experiences by comparing the two measures. Their results indicated that people often feel happier, healthier, and more relaxed after leisure travel (Chen et al., 2016). Concurrently, tourism industries pay attention to the challenging associations between tourism activities, their consequences, and the QOL of those involved in the producing consuming of tourism goods and services (Uysal et al., 2016).

However, the positive effects of travel appear short-lived. Several studies using a pre-post design (e.g., Nawijn, 2011; Nawijn et al., 2013) indicated that most people felt happier before and during their vacation; however, they were not happier after a vacation. In contrast, many people get inspired by their travel experience and want to travel again. Therefore, researchers need to consider both the type of travel and the experience acquired during travel to fully understand travel effectiveness on wellbeing.

### Factors of Wellbeing

It is also crucial to deepen understanding the relationships between people's wellbeing and social background factors. Income is most mentioned among the many social factors related to wellbeing, such as personal attributes and regional characteristics (e.g., Easterlin, 1974). Although income is a social background factor of wellbeing, it is also clear that there is no simple positive linear correlation between income and wellbeing. In particular, an association between income and subjective wellbeing is more likely to be positive when the average income person's material welfare accompanies rising income; that is, when people become more satisfied with their finances, they become more optimistic about their future (Diener et al., 2013).

Moreover, when asked to take stock of their lives, economically privileged people report being significantly more satisfied than their counterparts. However, when asked how happy they are now, the experiences of people with more assets do not differ significantly from those with fewer. Income has been reported as a moderately strong predictor of life evaluation but a weak predictor of positive and negative feelings (Diener et al., 2010). Similarly, Kahneman and Deaton (2010) concluded that having a low income is associated with low life evaluation and low emotional wellbeing. They are the frequency and intensity of experiences of joy, fascination, anxiety, sadness, anger, and affection that make one's life pleasant or unpleasant. Therefore, having a high income is associated with life satisfaction but not happiness.

Such indications that people cannot be happy by merely raising income have also influenced developed countries' policies. For instance, France proposed replacing conventional economic indicators (e.g., gross domestic product) with a happiness index as an economic situation indicator. The proposal illustrates the belief that a vision that emphasizes an increase in humans' happiness and the sustainability expansion of economic activities is necessary for human welfare in a country (Stiglitz et al., 2010).

### Experience Purchasing

Income is not the main factor influencing wellbeing. Dunn et al. (2011) suggested that people were often happier when they spent their money on experiences rather than things. Experiential purchases were “made with the primary intention of acquiring a life experience: an event or series of events that one lives through,” while material purchases were “made with the primary intention of acquiring a material good” (Van Boven and Gilovich, 2003). According to them, experiences make people happier because they are more open to positive reinterpretations, are a more meaningful part of one's identity, and contribute more to successful social bonds.

People seem to derive more happiness from experiences than from things because experiences are more likely to be shared with other people, and other people are our most cherished source of happiness (Dunn et al., 2011). Furthermore, Howell and Hill (2009) reported that experiential purchases represented money better spent more happiness to oneself and others.

Traveling during vacation would certainly mean investing money in the experience. Money is spent on various aspects during travel, such as accommodation, transportation, shopping, and food. Furthermore, most of the consumption other than shopping while traveling is in the form of experience. However, to our knowledge, little is known about the relationship between experience consumption and wellbeing in the tourism industry. Moreover, we can also understand the extent to which the amount of money spent on travel will influence subsequent wellbeing. In that case, there is a possibility of applying this information to effective policies in the tourism industry to improve people's subjective wellbeing and acquire more excellent profits. Therefore, we posited the following hypotheses:

Hypothesis 1a: People who traveled would exhibit higher wellbeing than those who did not.

Hypothesis 1b: People who spent more money during travel would exhibit higher wellbeing than those who did not.

### Recovery Experiences During Vacation and Wellbeing

Recovery can be defined as a process during which individual functional systems that have been called upon during a stressful experience return to their initial pre-stressor levels (Meijman and Mulder, 1998). While several studies suggested that vacation and travel improve wellbeing, Nawijn and Filep (2016) suggested that studies on tourism wellbeing must not only focus on the hedonic aspects of tourism or simple correlations between two variables but also analyses of more stringent causal relationships.

Recovery experiences are individual strategies devoted to restoring peoples' energy resources and maintaining their psychological and subjective wellbeing, which could help in stressful situations (Lee et al., 2016). There are four recovery experiences: control, detachment, mastery, and relaxation (e.g., Shimazu et al., 2012).

Control experience is defined as how people believe they can decide on something to do on their days off without being concerned about work and the household (Lee et al., 2016). The experience of control during leisure time could serve as a resource that enhances recovery from work while off work. This recovery occurs because it satisfies the desire for control and allows individuals to choose their preferred leisure activities (Shimazu et al., 2012).

Various studies have highlighted the concept of psychological detachment as a core dimension of recovery experiences in different organizations (Lee et al., 2016). Etzion et al. (1998) also conceptualized individuals' sense of distance from the work situation as psychological detachment.

Mastery refers to how people experience new and challenging events during their free time and gain a sense of achievement. Mastery experiences challenge individuals without overtaxing their capabilities but are not necessarily effortless, as they require some level of self-regulation (Sonnentag and Fritz, 2007).

Relaxation relates to how individuals relax during their free time (e.g., relaxing at home with friends or relatives). In an organizational setting, relaxation occurs when employees are allowed time off from work-related tasks, freeing them from the need to expend physical or mental effort (Tinsley and Eldredge, 1995). One of the advantages of relaxation in employees' recovery experiences is the potential to improve their wellbeing at work, which could help reduce sympathetic activation (Sonnentag and Fritz, 2007).

Out of the four recovery experiences, psychological detachment is most compatible with travel because travel is a behavior that moves people between different geographic locations (United Nations., 2008). According to Soldatenko and Backer (2019), novelty and escape from daily life were the most crucial push factors in deciding to take a vacation. Zhang and Peng (2014) reported that “experiencing something different” and “increasing my knowledge and experience” were essential motivations for Chinese respondents to travel to a foreign destination.

Kawakubo and Oguchi (2019) quantitatively investigated how Japanese employees' recovery experiences during vacation affected their wellbeing. They found that recovery experiences promoted employees' creativity and improved their occupational wellbeing and life satisfaction. Mastery was the most influential of the four elements of the recovery experience. Consequently, they speculated that Japanese people emphasize the experience of learning new skills and gaining knowledge while on vacation.

Based on the above discussion, among the four types of recovery experiences, we formulate hypotheses to verify whether psychological detachment and mastery are significant factors promoting wellbeing after a vacation:

Hypothesis 2a: Psychological detachment during vacation would promote wellbeing after the vacation.

Hypothesis 2b: Mastery experiences during vacation would promote wellbeing after the vacation.

This study was based on various previous studies and extended them to focus on experiences during a vacation. Although long-term data are difficult to obtain owing to procedural problems such as high cost and refusal to answer, longitudinal designs can provide better evidence of causality (Koys, 2001). Furthermore, they provide more evidence regarding the temporal sequence of variables and events than cross-sectional designs (Bullock et al., 1994).

This study, therefore, addressed the changes in wellbeing over time (including the vacation period) and examined factors that affect the said changes. We conducted multiple surveys before and after a winter vacation to achieve these aims. We expected to obtain some intriguing implications for what kind of vacation is more beneficial for people's wellbeing. The long-term success, sustainability, and competitiveness of tourism will undoubtedly depend on how it contributes to improving the QOL of all stakeholders (Uysal et al., 2016). If our hypothesized relationships are supported, it will generate evidence of the kind of experience most useful for vacationers. Additionally, tourism practitioners can improve tourism businesses by helping promote tourists' wellbeing through vacations.

## Methods

### Sample and Procedure

This study used an online survey enabling individuals from different regions (i.e., urban and rural areas) and age groups to participate. Respondents answered questionnaires according to instructions on the computer screen.

Participants completed the questionnaire at three time points: the first survey was conducted at the end of December 2017 (Time 1: before the vacation); the second survey was conducted immediately after the end of the winter vacation in early January 2018 (Time 2: immediately following the vacation); and the third survey was conducted in early February, 1 month after the second survey (Time 3: 1 month after the vacation).

Questionnaires were randomly distributed to the sample registered with the research company. They were controlled to stratify across ages (i.e., the 20's, 30's, 40's, 50's, and 60's) and to be equally portioned. The purpose was to ensure that age and gender effects did not occur as much as possible. At the same time, to prevent bias in the responses of the target population, we aimed to collect 500 responses in the end. There were 1,100 respondents in the first survey, 838 (76.18%) in the second survey, and 507 (46.09%) in the third survey. In this research, we targeted those who responded to all surveys. Thus, the total number of participants was 507 Japanese individuals from the general population (255 men and 252 women).

### Measures

Questionnaires consisted of items seeking demographic information (e.g., gender, age, education status, and household income) and details on how the respondents spent their winter vacation (e.g., foreign travel, domestic travel, homecoming, or staying home). We also sought information on travel expenses by entering the actual cost (e.g., accommodation, transportation, food, and shopping while traveling). In addition to the items mentioned above, we used the following psychological measures.

#### Recovery Experience

Respondents' recovery experiences during the winter vacation were assessed using the Recovery Experience Scale (Sonnentag and Fritz, 2007). The 15-item scale has four dimensions: (1) psychological detachment, (2) relaxation, (3) mastery, and (4) control. Responses are rated on a 5-point Likert-type scale (1 = *strongly disagree* to 5 = *strongly agree*).

#### Life Satisfaction

Life satisfaction refers to an overall evaluation of the quality of one's life (Pavot and Diener, 1993). We assessed participants' life satisfaction using the Satisfaction with Life Scale (SWLS; Diener et al., 1985). The SWLS is a short five-item instrument designed to measure global cognitive judgments of satisfaction with one's life. The items were measured using a Likert-type scale ranging from 1 (*strongly disagree*) to 7 (*strongly agree*).

#### Wellbeing

We adopted the PERMA-profiler (Butler and Kern, 2016) to measure changes in participants' wellbeing through the winter vacation in five pillars: positive emotion, engagement, relationships, meaning, and accomplishment. The PERMA-profiler was developed to apply measurement changes in wellbeing at the individual, community, and national levels (Butler and Kern, 2016). We used the summary score of the 15 items as an indicator of the participants' general wellbeing following (Smith et al., 2021).

To construct the questionnaires, we used the PERMA-profiler for all three surveys. In contrast, the Recovery Experiences Scale was used in the second survey immediately after the vacation to verify them during the winter vacation. Other scales and demographic items were evaluated once in the first survey. Taken together, at Time 1, we provided the PERMA-profiler and demographic items. At Time 2, we provided the PERMA-profiler and the Recovery Experiences Scale. At Time 3, we provided the PERMA-profiler.

## Findings

The following analyses were conducted using SPSS version 23 (Windows).

### Descriptive Statistics and Correlation Analysis

The sample was divided into two based on gender, with an average age of 45.03 years (*SD* = 13.40; range = 20–69 years). Most respondents had completed higher education (college: 52.3%; graduate school: 8.7%).

Means, standard deviations, and correlations between the variables are presented in Table 1. Psychological detachment, relaxation, mastery and control in Table 1 are components of the recovery experience. Participants' wellbeing 1 month after the winter vacation (i.e., Wellbeing Time 3) showed significant positive correlations with age (*r* = 0.22, *p* < 0.01), education (*r* = 0.17, *p* < 0.01), household income (*r* = 0.17, *p* < 0.01), life satisfaction (*r* = 0.66, *p* < 0.01), psychological detachment (*r* = 0.18, *p* < 0.01), relaxation (*r* = 0.26, *p* < 0.01), mastery (*r* = 0.38, *p* < 0.01), and control (*r* = 0.28, *p* < 0.01).

**Table 1:** Means, standard deviations, and correlation coefficients between research variables.

	**Variable**	***M***	***SD***	**1**	**2**	**3**		**4**		**5**		**6**		**7**		**8**		**9**		**10**		**11**		**12**
1	Gender			—	0.00	−0.20	**	−0.52	**	0.01		0.01		0.00		0.05		0.00		0.03		−0.01		0.03	
2	Age years	45.03	13.4		—	−0.01		0.10	*	0.15	**	0.20	**	0.21	**	0.22	**	0.02		0.09	*	0.05		0.14	**
3	Education					—		0.31	**	0.14	**	0.15	**	0.19	**	0.17	**	0.03		0.02		0.17	**	0.05	
4	Household income							—		0.22	**	0.22	**	0.23	**	0.17	**	0.10	*	0.09		0.17	**	0.10	*
5	Life satisfaction	19.25	6.36							—		0.71	**	0.66	**	0.66	**	0.12	**	0.13	**	0.27	**	0.14	**
6	Wellbeing time 1	85.59	22.70									—		0.82	**	0.81	**	0.17	**	0.26	**	0.38	**	0.26	**
7	Wellbeing time 2	83.22	23.10											—		0.92	**	0.21	**	0.31	**	0.41	**	0.32	**
8	Wellbeing time 3	83.72	23.59													—		0.18	**	0.26	**	0.38	**	0.28	**
9	Psychological detachment	12.82	4.23															—		0.68	**	0.39	**	0.40	**
10	Relaxation	14.71	3.62																	—		0.34	**	0.61	**
11	Mastery	10.91	4.03																			—		0.41	**
12	Control	14.85	3.48																					—	

** *p < 0.01*,

* *p < 0.05. Time 1: before the vacation, Time 2: immediately following the vacation, Time 3: 1month after the vacation*.

*Gender: 1 = Male, 2 = Female; Education: 1 = Junior high school, 2 = High school, 3 = Professional school, 4 = Junior College, 5 = College, Graduate school; Household Income (in Japanese Yen): 0 = No income, 1 = Under ¥1000000, 2 = ¥2000000–2999999, 3 = ¥3000000–3999999, 4 = ¥4000000–4999999, 5 = ¥5000000–5999999, 6 = ¥6000000–6999999, 7 = ¥7000000–7999999, 8 = ¥8000000–8999999, 9 = ¥9000000–9999999, 10 = ¥10000000–14999999, 11 = ¥15000000–19999999, 12 = More than ¥20000000. Higher scores on life satisfaction, wellbeing, psychological detachment, relaxation, mastery, and control indicate greater levels of the construct being measured*.

### Group Selection

To verify the effects of the winter vacations on wellbeing, we divided participants into those who traveled abroad or domestically. Additionally, during the New Year holidays in Japan, many people return home to visit friends and relatives, although they may stay in different types of accommodations. Therefore, we added homecoming as a typical holiday behavior during the study period. Moreover, we created one group that included people who did not travel anywhere. The survey was conducted such that the above four groups had almost the same number of people.

The respondents were divided into four groups based on their travel: 89 were assigned to the foreign travel group, 156 to the domestic travel group, 115 to the homecoming group, and 147 to the staying-home group.

Before conducting analyses using these groups, we accounted for any difference in each group's demographic characteristics. The chi-square tests on gender (χ^2^[3] = 0.38, ns.), education (χ^2^[12] = 39.07, *ns*.), and household income (χ^2^[15] = 21.08, *ns*.) revealed no significant differences between the groups. Thus, subsequent analyses were conducted without considering the influence of the above demographic factors.

Moreover, to confirm an adequate sample size, we conducted a power analysis using G^*^Power 3 software (Faul et al., 2007). Based on our group settings [i.e., power (1 – β = 0.80) with low to medium effect size (f = 0.20) at the significance level α = 0.05; the number of groups was four], the minimum required sample size was 280. Thus, we considered that the sample size was sufficient.

### Comparison of Wellbeing Between Groups Before and After Winter Vacation

Before comparing the four groups, we conducted a pre-analysis comparison of the groups that traveled (i.e., foreign travel and domestic travel) and the groups that did not (i.e., homecoming and staying home). Independent samples *t*-tests revealed that, at all times, the wellbeing of the group that traveled (*M*_Time1_ = 88.99, *SD*_Time1_ = 22.45; *M*_Time2_ = 86.31, *SD*_Time2_ = 21.86; *M*_Time3_ = 86.42, *SD*_Time3_ = 22.28) was higher than that of those who did not travel (*M*_Time1_ = 82.41, *SD*_Time1_ = 22.52; *M*_Time2_ = 80.33, *SD*_Time2_ = 23.88; *M*_Time3_ = 81.19; *SD*_Time3_ = 24.52; Time 1: *t* (505) = 3.29, *d* = 0.29, *p* < 01; Time 2: *t* (505) = 2.93, *d* = 0.26, *p* < 01; Time 3: *t* (505) = 2.51, *d* = 0.22, *p* < 05).

To compare the groups in more detail, we performed a 4 × 3 Group (foreign travel, domestic travel, homecoming, and staying home) × Time (Time 1, Time 2, and Time 3) repeated-measures analysis of variance (ANOVA) for the subjective happiness of vacationers. For the total PERMA-profiler score, significant main effects of the group were observed for group [*F* (3, 503) = 5.16, *p* < 0.01; $\eta_{\text{p}}^{2}$ = 0.03] and time [*F* (2, 1006) = 9.79, *p* < 0.01; $\eta_{\text{p}}^{2}$ = 0.02]. However, there was no significant interaction between group and time [*F* (6, 1006) = 0.90, *ns*.; $\eta_{\text{p}}^{2}$ = 0.01].

**Figure 1:**
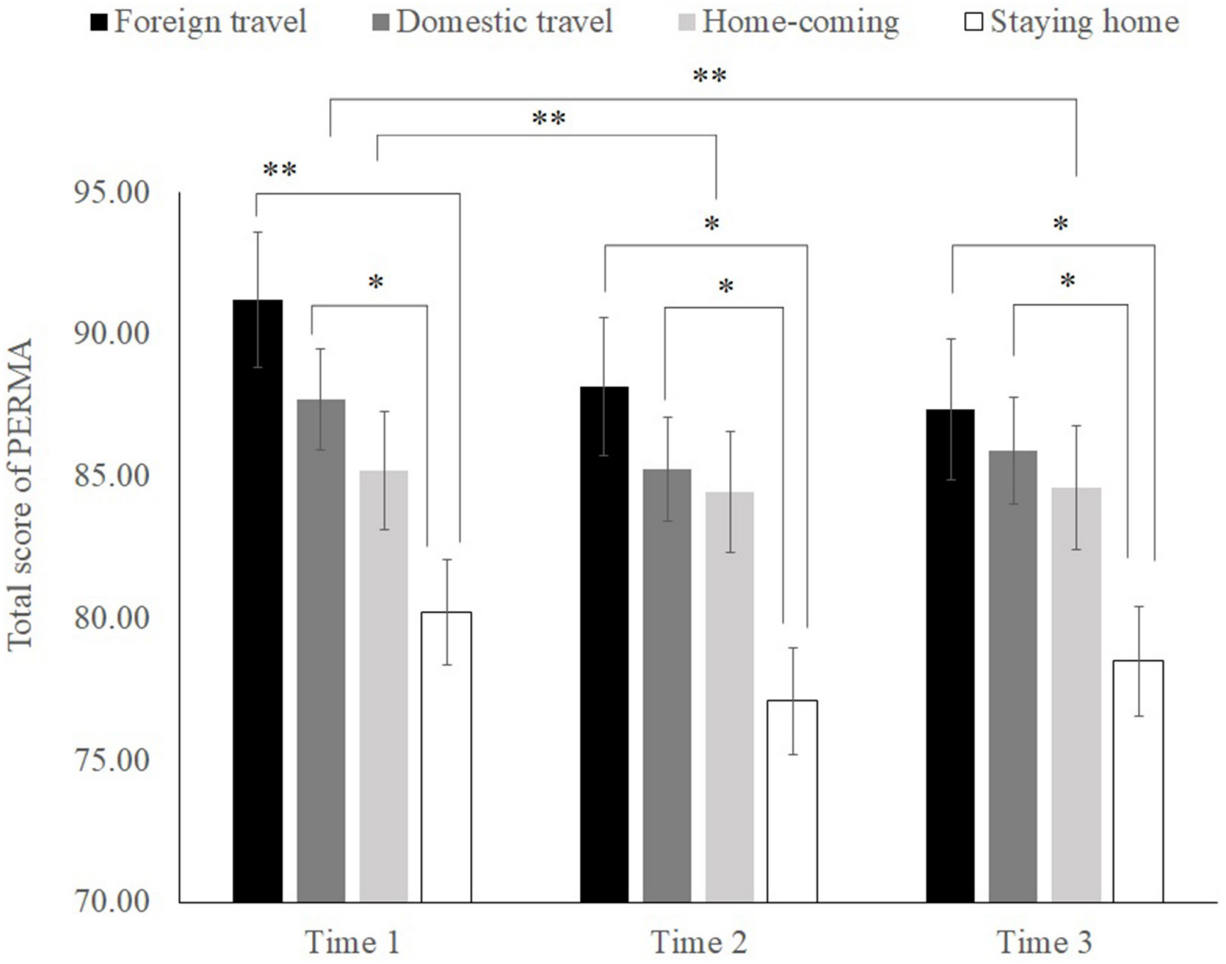
Differences in total PERMA scores between groups. *p < 0.05, **p < 0.01. The error bars represent standard errors of measurement. Time 1: before the vacation, Time 2: immediately following the vacation, Time 3: 1 month after the vacation.

Analysis of simple main effects confirmed that the scores of the foreign and domestic travel groups (*M*_foreign_ = 88.92, *SD*_foreign_ = 24.00; *M*_domestic_ = 86.28, *SD*_domestic_ = 21.10) were significantly higher (*p* < 0.05) than those of the staying-home group (*M* = 78.61, *SD* = 24.02). Regarding the effects of time, the scores of the groups at Time 1 were significantly higher (*p* < 0.01) than those of the groups at Times 2 and 3 (Figure 1). This result suggests that people who traveled during the winter vacation, regardless of whether their destination was overseas or domestic, had higher wellbeing than those who did not. Thus, Hypothesis 1a was supported.

Subsequently, we examined the relationship between travel expenses and wellbeing. We included only participants who traveled in this analysis because the analysis was based on the amount spent on the travel. Participants reported spending ¥3,206.55 (*SD* = 5334.73) on foreign travel and ¥804.22 (*SD* = 1100.55) on domestic travel on average. Concerning the relationship with annual income, 50% of the respondents spent approximately 2.33% of their annual income on travel during winter.

Further, after controlling for household income, we calculated partial correlations to examine the relationships between the influence of consumption expenditure and travel. We controlled for annual income because participants with higher annual incomes could spend larger money on travel. However, there were no significant relationships with wellbeing at Time 1 (*r* = −0.03, *ns*.), Time 2 (*r* = −0.10, *ns*.), and Time 3 (*r* = −0.04, *ns*.). Thus, Hypothesis 1b was not supported.

### Comparison of Wellbeing Between the Four Types of Vacation

We undertook Confirmatory factor analysis (CFA) to confirm the constructs of the Recovery experiences scale (Sonnentag and Fritz, 2007). In our analyses, three standards of goodness-of-fit were used to check how well each CFA model fit the data. We considered CFI > 0.90 (Bentler and Bonett, 1980), RMSEA < 0.08 (Browne and Cudeck, 1993), and SRMR < 0.08 (Hu and Bentler, 1998) as representing acceptable model fit. The results of the CFA showed that the measurement model fit the data well, and the goodness-of-fit indices were in line with the established criteria (see Table 2; CFI = 0.937, RMSEA = 0.073, SRMR = 0.069).

**Table 2:** Confirmatory factor analysis result of the recovery experience scale.

					**Bootstrap**	
					**95% Cl**	
**Variables (Cronbach's α)**		***M***	***SD***	**Standardized factor loading**	**Lower**	**Upper**	**CR**	**AVE**
Psychological detachment (0.87)							0.88	0.64
PD1		3.25	1.26	0.81	0.77	0.85		
PD2		2.92	1.32	0.71	0.65	0.76		
PD3		3.29	1.24	0.84	0.80	0.87		
PD4		3.41	1.17	0.83	0.79	0.86		
Relaxation (0.89)							0.91	0.72
RE1		3.78	1.03	0.80	0.75	0.84		
RE2		3.70	1.05	0.91	0.88	0.94		
RE3		3.67	1.05	0.91	0.87	0.93		
RE4		3.41	1.17	0.77	0.72	0.81		
Mastery (0.89)							0.89	0.66
MA1		2.84	1.20	0.80	0.75	0.83		
MA2		2.58	1.13	0.84	0.79	0.88		
MA3		2.61	1.15	0.82	0.78	0.86		
MA4		2.88	1.17	0.80	0.76	0.84		
Control (0.85)							0.85	0.59
CO1		3.74	1.11	0.74	0.69	0.79		
CO2		3.74	1.09	0.83	0.78	0.87		
CO3		3.75	1.08	0.83	0.80	0.87		
CO4		3.58	0.99	0.67	0.59	0.74		

*Cl, confidence interval; CR, composite reliability; AVE, average variance extracted*.

Subsequently, we examined the differences in the recovery experience scores among the four groups mentioned above. The four facets of recovery experiences (i.e., psychological detachment, relaxation, mastery, and control) were significantly correlated with one another (Table 1). Therefore, we ran a multivariate ANOVA with the four facets of recovery experiences as the dependent variables and group as the independent variable.

**Figure 2:**
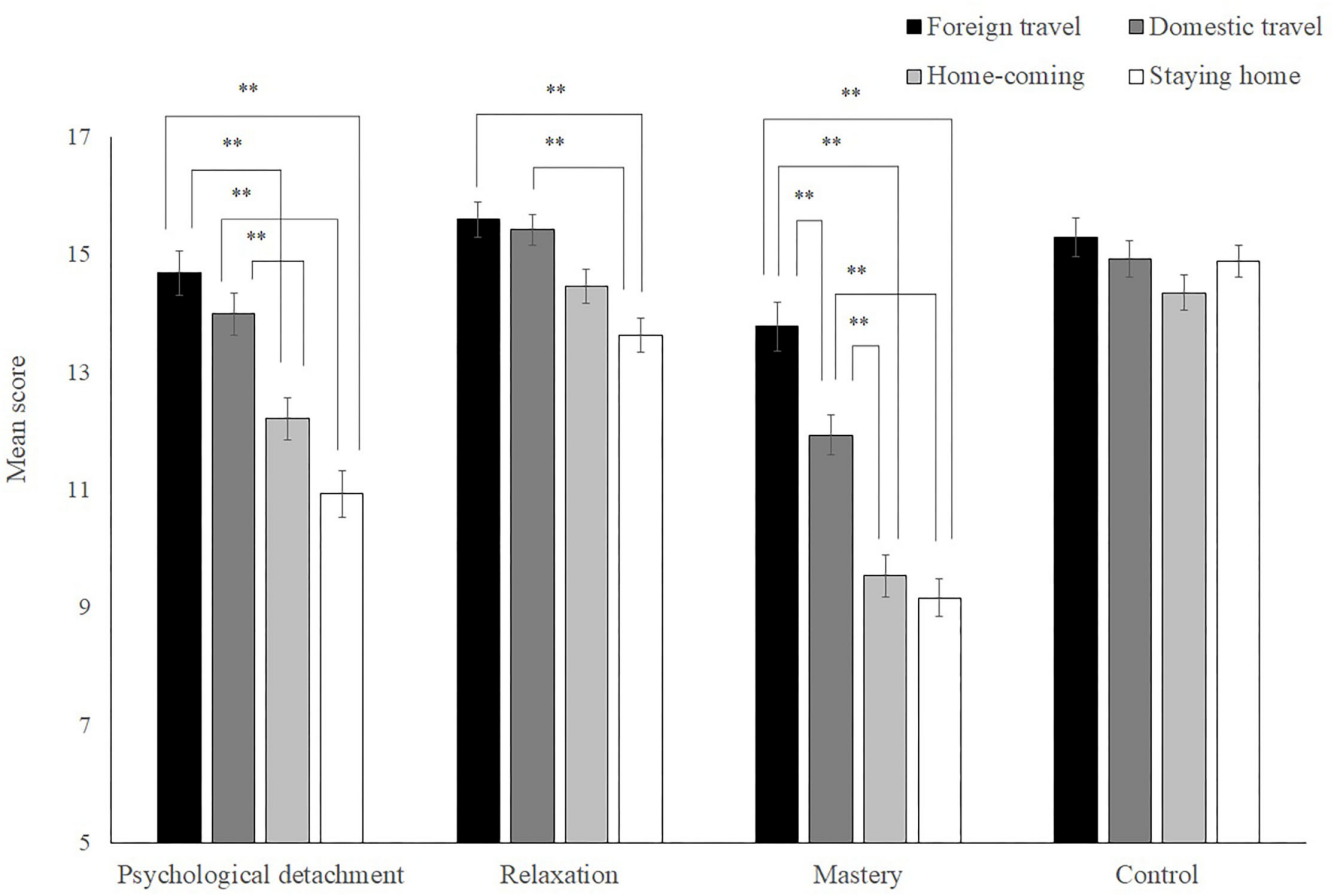
Differences in the mean score of recovery experiences between groups. **p < 0.01. The error bars represent standard errors of measurement.

The results revealed that the multivariate main effects of group were significant [Wilks's Λ = 0.721, *F* (12, 1323) = 13.18, *p* < 0.01, $\eta_{\text{p}}^{2}$ = 0.05]. There were also significant univariate main effects of group for psychological detachment [*F* (3, 503) = 22.88, *p* < 0.01; $\eta_{\text{p}}^{2}$ = 0.12], relaxation [*F* (3, 503) = 8.72, *p* < 0.01; $\eta_{\text{p}}^{2}$ = 0.05], and mastery [*F* (3, 503) = 39.28, *p* < 0.01; $\eta_{\text{p}}^{2}$ = 0.19], but not for control [*F* (3, 503) = 1.30, *ns*; $\eta_{\text{p}}^{2}$ = 0.015; Figure 2]. Pairwise comparisons using a Bonferroni correction indicated significant differences in the three types of experience scores between the foreign travel group, the domestic travel group, and the other two groups.

### Influences of Recovery Experiences on Wellbeing 1 Month After Winter Vacation

Hierarchical regression analyses were conducted to examine the relationship between independent variables and vacationers' wellbeing 1 month after winter vacation (Wellbeing Time 3). To detect the effect of recovery experience on wellbeing, we controlled for demographic factors. In the first step, demographic factors (i.e., gender and age) were included. The second step added the sociodemographic characteristics of the respondents (i.e., household income and education). In the third step, life satisfaction was added. Finally, in the fourth step, we added the four sub-factor scores (i.e., psychological detachment, relaxation, mastery, and control) of the recovery experience (Table 3).

**Table 3:** Results of the hierarchical regression analysis for wellbeing (Time 3).

				**95% Cl**		
**Predictor**	**β**	***SE***	***t***	**Lower**	**Higher**	***ΔR*** **^2^**
Step 1						
Sex	0.70	2.14	0.33	−3.50	4.90	
Age	0.38	0.08	4.72^**^	0.22	0.53	
Step 2						0.05^**^
Household income	1.43	0.42	3.40^**^	0.60	2.25	
Education	2.21	0.81	2.71^**^	0.61	3.81	
Step 3						0.36^**^
Life satisfaction	2.35	0.13	17.56^**^	2.09	2.61
Step 4						0.05^**^
Psychological detachment	−0.31	0.26	1.20	−0.82	0.20
Relaxation	0.63	0.34	1.87	−0.03	1.30	
Mastery	0.92	0.23	4.06^**^	0.48	1.37	
Control	0.52	0.30	1.78	−0.06	1.11	
Model *R*^2^ = 0.49, *F*(9, 459) = 52.48, *p* <0.001					

** *p < 0.01*.

In the final regression model, the nine included variables explained a significant proportion of the variance in the dependent variable (Table 3). Age, household income, and education significantly affected vacationers' wellbeing. Further, life satisfaction had a significant positive influence. In contrast, when we examined the influences of the experience during the winter vacation in the fourth step, only mastery was found to have a significant influence. Thus, the results only supported Hypothesis 2b. The above series of analyses revealed that mastery was the most essential among the four recovery experiences.

## Discussion

We conducted surveys at different time points to identify the changes in wellbeing before and after a winter vacation. The primary objective was to capture and examine the factors that create a more effective vacation based on the experiences during the participants' winter vacation.

Our ANOVA revealed significant main effects for both group and time regarding the effects of travel during vacations. The differences between the groups were clarified. People who traveled during vacation exhibited higher wellbeing than those who stayed home. This result indicates that getting away from daily life during long holidays and experiencing something out of the ordinary improved vacationers' wellbeing.

Subsequently, we divided the survey participants into four groups based on the type of travel to examine the difference in wellbeing and recovery experience. The results suggested that foreign and domestic travelers' wellbeing was higher than those who stayed home. Concurrently, the three recovery experiences other than control were also higher in the last two groups. These results may indicate the superior effects of investing money in wellbeing experiences, as noted in previous studies (e.g., Van Boven and Gilovich, 2003; Dunn et al., 2011).

Traveling requires a relatively large amount of money compared to being at home on vacation. However, the current study showed that expenses during travel were not associated with wellbeing. What can be derived from this result is that investing much money on vacation was not a shortcut to promote wellbeing. Thus, as it is not merely spending too much money on experiences, providing a “valuable experience” will also challenge the tourism industry.

In contrast, when focusing on the change in wellbeing from Time 1 to Time 3, our results provided sufficient evidence to indicate that wellbeing gradually decreased in these periods. Specifically, wellbeing before or during the winter vacation was the highest, gradually declined immediately after the vacation, and was the lowest 1 month later. Based on this point, we emphasized the experiences or factors that significantly influenced wellbeing after the vacation in subsequent analyses.

It is undoubtedly crucial to consider social factors such as income or education and preliminary life satisfaction when discussing wellbeing. However, even though we controlled for such factors, the mastery experience of a vacation significantly impacted vacationers' wellbeing. Mastery experiences refer to activities performed during the time off work, which distract from the job by providing challenging experiences and learning opportunities in other domains and offer opportunities to experience competence and proficiency (Sonnentag and Fritz, 2007). Apart from the current results, another study has also found that tourists from the high-income group had a greater interest in engaging in fun activities related to self-development (Zhang and Peng, 2014). People place a high value on learning new skills and knowledge and hard-won experiences while on vacation, and such experiences may help improve wellbeing.

Vacations allow people to reflect on their lives and the benefits gained from travel to be transferred back into their everyday lives in new relationships, abilities, and changed perspectives (Packer and Gill, 2017). Understanding the relationship between improved wellbeing from vacations and promoting factors can help the tourism industry design and implement promotional materials that target specific customer segments.

This research provides new ideas for tourism and wellbeing. Our results suggest that it is necessary to travel and enjoy recovery experiences while on leave from work or daily duty. How can people enhance savoring their vacation, as so many would like to do? First, the vacation needs to be enjoyable – people generally prefer to experience positive rather than negative emotions across cultures (Kuppens et al., 2008).

Moreover, according to Bryant and Veroff (2007), one strategy that enhances opportunities to savor life is to take “time-outs” from ordinary, ongoing life purposefully. Once there is time to savor life, a person must remain open to various savoring experiences. People involved in the tourism industry should be conscious of these points.

This study confirmed the importance of mastery experiences during a vacation and determined factors related to wellbeing. The findings of this study could apply to the tourism and hospitality industries. According to Sirgy et al. (2017), any leisure activity should be designed to provide benefits related to basic needs such as safety, health, economics, sensory, escape, and sensation or stimulation. In addition to meeting basic needs, the leisure activity should also be designed to provide benefits related to growth needs, such as the self, aesthetics, mastery, relatedness, and distinctiveness.

The tourism industry should encourage people to take vacations by presenting and emphasizing its benefits, such as promoting vacationers' curiosity and knowledge acquisition. We recommend providing activities that can help customers experience something new and challenging. Considering the characteristics of the mastery experience (i.e., the extent to which people experience new and challenging events during their free time and gain a sense of achievement), activities that people cannot experience in everyday life, and those that also pose some degree of difficulty may be preferable. Tourism service providers should consider providing various activities for their customers. In this way, vacationers could select and make decisions about their behavior during their vacation, leading to control experiences. While there is no doubt that many tourist destinations already offer various activities and relaxing environments, tourism organizations and businesses can attract even more tourists from specific groups by designing more relevant products and promoting them by highlighting sought-after features.

In summary, while several scholars have shown vacations as profitable opportunities, we demonstrated that the perceived experiences were essential in determining subsequent wellbeing after the vacations. The results showed that people who traveled during winter vacation had higher subjective wellbeing than those who did not. We confirmed that mastery experiences that provide challenges and learning opportunities are valid within diverse vacation experiences. These results elucidate the question of what kind of vacation is more effective. This study also suggested that tourism practitioners could better manage their customers' experience during their vacations, and these efforts will arguably contribute not only to the wellbeing of vacationers but also to the tourism industry.

## Data Availability Statement

The raw data supporting the conclusions of this article will be made available by the authors, without undue reservation.

## Ethics Statement

The studies involving human participants were reviewed and approved by College of Contemporary Psychology, Rikkyo University. The patients/participants provided their written informed consent to participate in this study.

## Author Contributions

AK analyzed data and wrote the first draft of the manuscript. TO supervised the project and critically reviewed the manuscript. Both authors contributed to manuscript revision, read, and approved the submitted version.

## Funding

This research was supported by the Private University Research Branding Project (Inclusive Academics: Interdisciplinary research into “health and diversity” of humans and animals) and JSPS KAKENHI Grant No. JP20H04444.

## Conflict of Interest

The authors declare that the research was conducted in the absence of any commercial or financial relationships that could be construed as a potential conflict of interest.

## Publisher's Note

All claims expressed in this article are solely those of the authors and do not necessarily represent those of their affiliated organizations, or those of the publisher, the editors and the reviewers. Any product that may be evaluated in this article, or claim that may be made by its manufacturer, is not guaranteed or endorsed by the publisher.
